# 非小细胞肺癌患者胸腔镜及传统开胸手术前后甲状腺激素变化的比较

**DOI:** 10.3779/j.issn.1009-3419.2013.12.06

**Published:** 2013-12-20

**Authors:** 文鑫 田, 宏峰 佟, 耀光 孙, 新 李, 青峻 吴, 超 马, 鹏 焦

**Affiliations:** 1 100730 北京，卫生部北京医院胸外科 Department of Toracic Surgery, Beijing Hospital, Beijing 100730, China; 2 100730 北京，卫生部北京医院核医学科 Department of Nuclear Medicine, Bejing Hospital, Beijing 100730, China

**Keywords:** 胸腔镜手术, 甲状腺激素, 肺肿瘤, Video-assisted thoracoscopic surgery, Tyroid hormones, Lung neoplasms

## Abstract

**背景与目的:**

电视辅助胸腔镜手术（video-assisted thoracoscopic surgery, VATS）是胸部微创手术的代表，相对于传统开胸手术（traditional open surgery, TOS），VATS具有创伤小、疼痛轻、恢复快等优势。本研究旨在比较VATS与传统开胸肺叶切除治疗非小细胞肺癌（non-small cell lung cancer, NSCLC）围手术期甲状腺激素水平的变化。

**方法:**

选取卫生部北京医院胸外科2010年10月-2012年8月收治的NSCLC患者44例，根据手术方式的不同分为：胸腔镜组（VATS组，25例）及传统开胸组（TOS组，19例）。两组于手术前1天、术后第1天、2天、3天、7天分别测定游离T3（free T3, FT3）、游离T4（free T4, FT4）、反T3（reverse T3, rT3）、促甲状腺激素（thyroid stimulating hormone, TSH）的水平，观察两组术后各指标的变化规律，比较两组间变化的差异。

**结果:**

术前患者甲状腺激素水平均在正常值范围内，两组比较无统计学差异（*P*>0.05）。FT3及TSH术后均表现为先降低后升高，分别于术后第3天、术后第1天达到最低水平，术后第7天时VATS组已恢复至术前水平，但TOS组FT3仍明显低于术前（*P*=0.032）；FT4及rT3术后均表现为先升高后降低，均于术后第2天达到峰值，TOS组术后第1天、2天、3天rT3水平均明显高于VATS组（*P* < 0.05）。术后FT3降低及rT3升高均超出正常值范围，而FT4及TSH变化均在正常值范围内。两组间各指标变化趋势比较，rT3变化有统计学差异（*F*=7.557, *P*=0.009）。

**结论:**

NSCLC患者行肺叶切除术后均出现正常甲状腺功能病态综合征，相对于传统开胸手术，胸腔镜手术对机体甲状腺激素水平影响小，机体应激反应小，利于肺癌患者术后恢复。

## Abstract (en)

**Background and objective:**

Video-assisted thoracoscopic surgery (VATS) is the representative thoracic minimally invasive surgery. Compared with traditional open surgery (TOS), VATS has an advantage of less invasiveness, quicker recovery and milder postoperative pain. The aim of this study is to compare the influences of VATS and conventional thoracotomic lobectomy on thyroid hormones in treatment for non-small cell lung cancer (NSCLC).

**Method:**

From Oct. 2010 to Aug. 2012, 44 consecutive patients with NSCLC were recruited and divided into two groups: VATS group (25 patients) and TOS group (19 patients). All the patients were drawn blood to measure the plasma levels of free T3, free T4, reverse T3 and TSH on the morning of the day before surgery, the postoperative day (POD)1, POD2, POD3 and POD7. We described the perioperative variation trend of the thyroid hormones and compared the differences between VATS group and TOS group.

**Results:**

There were no differences of thyroid hormone levels between the two groups before surgery. After surgery, the levels of FT3 and TSH were first dropped, then elevated and reached the lowest level on POD3 and POD1 separately. On POD7, they regained the before-surgery levels in VATS group while FT3 level was still significantly lower in TOS group (*P*=0.032). The FT4 and rT3 levels were first elevated, and then dropped after surgery. They both reached the peak concentrations on POD2, and rT3 levels of TOS group were significantly higher than those of VATS group on POD1, POD2 and POD3 (*P* < 0.05). The changes of FT3 and rT3 levels were beyond normal ranges while changes of FT4 and TSH levels were within normal ranges. When variation trend of all the thyroid hormones were compared between the two groups, only rT3 level was found to have significant statistical differences (*F*=7.557, *P*=0.009).

**Conclusion:**

All NSCLC patients after lobectomy have Euthyroid sick syndrome (ESS). Compared with traditional thoracotomy, VATS surgery has smaller influence on perioperative thyroid hormones and demonstrates a weaker acute stress reaction, which can benefit postoperative recovery of NSCLC patients.

电视辅助胸腔镜手术（video-assisted thoracoscopic surgery, VATS）是胸部微创手术的代表，与传统开胸手术（traditional open surgery, TOS）相比，它具有创伤小、出血少、恢复快、疼痛轻、住院时间短等优势^[1-3]^，已被越来越广泛地应用到各种胸部疾病，特别是早期肺癌的治疗。已有研究^[4, 5]^表明，VATS肺叶切除治疗早期肺癌术后患者能获得更高的生活质量。本研究旨在通过前瞻性的对照实验，比较VATS与传统开胸手术后机体甲状腺激素变化的异同及临床意义。

## 1 研究对象和方法

### 1.1 研究对象

2010年10月-2012年8月，卫生部北京医院胸外科住院的非小细胞肺癌（non-small cell lung cancer, NSCLC）患者，术前评估无开胸或VATS手术禁忌，拟行肺叶切除、系统性淋巴结清扫手术的患者为待入组者。若手术最终为肺叶切除+系统性淋巴结清扫，且术前术后不存在影响甲状腺激素水平情况的患者为最终入组患者。

入组标准：①肿块 < 6 cm，叶支气管未受侵犯；②行肺叶切除+系统性淋巴结清扫手术；③术前术后不存在影响甲状腺激素水平的疾病或其他情况；④无远处转移。

根据手术方式不同，入组患者分为胸腔镜手术组（VATS组）和传统开胸手术组（TOS组）。

### 1.2 手术方法

手术中，所有患者均行静脉吸入复合全麻，双腔气管插管，术中均行单肺通气，每个患者均行解剖性肺叶切除加系统性肺门纵隔淋巴结清扫术。

VATS肺叶切除：手术切口由主操作孔、腔镜孔及一个辅助操作孔组成，其中主操作孔切口长约3 cm-5 cm，选用腋前线第4或5肋间进胸，腔镜孔选用腋中线第7或8肋间进胸，辅助操作孔选择肩胛线第7或8肋间。不使用肋骨牵开器，根据情况选用乳突牵开器牵开皮肤肌层或切口保护套。胸内操作完全在镜下完成，支气管、肺裂及较大血管的处理采用一次性切割缝合器，较小血管处理采用丝线结扎或缝扎。

TOS肺叶切除：一般选用第5或6肋间后外侧切口进胸，切口长约15 cm-20 cm，胸壁肌肉切断，使用肋骨牵开器牵开肋骨，胸内操作完全为直视下完成，血管、支气管及肺的处理采用切割缝合器，或丝线结扎、缝合。

系统性淋巴结清扫：至少4站淋巴结，其中包括肺门淋巴结在内的肺门肺内淋巴结至少1站，包括隆突下淋巴结在内的纵隔淋巴结至少3站。

### 1.3 材料与仪器

FT3、FT4、TSH化学发光酶免试剂盒，rT3放射免疫试剂盒，均由北京北方生物技术研究所提供。γ计数器（北京核仪器厂，BH6020型组合式γ计数器）、离心机、振荡机、水浴箱、冰箱、微量加样器等。

### 1.4 实验指标测定方法

分别于术前1天，术后第1天、2天、3天、7天清晨，空腹抽取静脉血5 mL，3, 000 r/min离心5 min后取血浆储存于-40 ℃冰箱中。待血样收集完成后，分别用化学发光酶免疫分析法测定FT3、FT4、TSH的水平，放免试剂盒测量rT3的水平。各激素浓度测定时严格按照试剂盒说明书进行操作。

### 1.5 统计学分析方法

使用软件SPSS 17.0做数据统计学分析，两组人群资料差异用*χ*^2^检验或两独立样本*t*检验，两组间不同时间点FT3、FT4、rT3、TSH水平差异分析采用两独立样本*t*检验或者重复测量数据的方差分析。*P* < 0.05为差异具有统计学意义。

## 2 结果

本研究共44例患者入组，分为VATS组25例和TOS组19例，两组资料见表 1。两组在年龄、性别、术前FEV_1_值、手术时间、肿瘤直径、淋巴结清扫个数、站数等方面无统计学差异（*P*>0.05）。VATS组1例患者术后出现肺漏气，予高渗葡萄糖胸腔内注射，于术后第9天顺利拔除胸管出院；1例于术后第3周左右出现乳糜胸，予以再次开胸手术结扎胸导管后治愈。

**1:** VATS及TOS组患者临床及手术资料 Clinical and surgical characteristics of patients of VATS and TOS group

Characteristic	VATS group	TOS group	*P*
Numbers	25	19	
Age (year)	61.56±10.20	62.58±10.70	NS
Sex (Male/Female)	15/10	10/9	
FEV_1_ (L)	2.49±0.68	2.54±0.63	NS
Tumor diameter (cm)	3.52±1.17	3.83±1.83	NS
Duration of surgery (min)	160.60±48.03	209.60±81.8	NS
Tumor location			
LUL	5	9	
LLL	5	6	
RUL	11	2	
RML	2	0	
RLL	2	2	
Histology			
Adenocarcinoma	18	14	
Squamous cell carcinoma	6	5	
Large cell carcinoma	1	0	
TNM stage			
Ⅰ	18	10	
Ⅱ	2	4	
Ⅲa	5	5	
Numbers of lymph nodes dissected	19.60±9.20	15.90±8.80	NS
Groups of lymph nondes dissected	5.16±1.03	4.84±1.12	NS
LUL: left upper lobe; LLL: left lower lobe; RUL: right upper lobe; RML: right middle lobe; RLL: right lower lobe; NS indicates lack of significant differences; VATS: video-assisted thoracoscopic surgery; TOS: traditional open surgery.			

围手术期甲状腺激素水平的变化趋势及两组间比较（图 1）。术前两组各甲状腺功能指标均在正常值范围内，两组比较无统计学差异（*P*>0.05）。FT3水平两组术后均呈现先降低后升高的变化规律，于术后第3天达到最低水平，后逐渐升高，术后第7天时已恢复至正常值范围，术后第7天与术前比较，VATS组已无统计学差异（*P*=0.057），TOS组仍明显低于术前（*P*=0.032）。FT4水平两组术后均呈现先升高后降低的变化规律，于术后第2天达到峰值，之后下降，术后第3天已恢复至术前水平（*P*>0.05），术后第7天时较前略升高。rT3水平术后两组均呈现先升高后降低的变化规律，术后第2天达到峰值，后逐渐下降，术后第7天时已接近正常值范围，但仍高于术前水平（*P* < 0.05）。术后第1天、2天、3天TOS组rT3水平均明显高于VATS组（*P* < 0.05）。TSH水平两组术后均呈现先下降后升高的变化规律，于术后第1天达到最低水平，后逐渐升高，术后第7天时已恢复至术前水平。FT3、rT3的变化均超出正常值范围，FT4、TSH的变化均在正常值范围内。两组间各指标变化趋势比较，仅rT3的变化两组间有明显统计学差异（*F*=7.557, *P*=0.009），余变化差异无统计学意义（*P*>0.05）。

**1:**
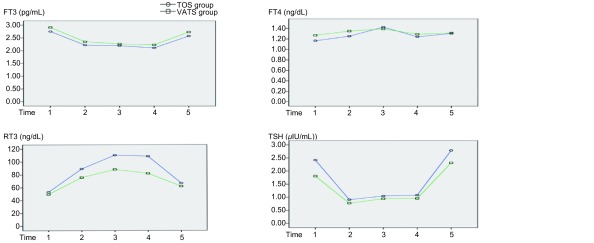
围手术期VATS组及TOS组甲状腺激素变化的比较。Time 1：术前1天；Time 2：术后第1天；Time 3：术后第2天；Time 4：术后第3天；Time 5：术后第7天。各甲状腺功能指标正常值范围：FT3：2.3 pg/mL-4.2 pg/mL；FT4：0.89 ng/dL-1.76 ng/dL；TSH：0.35 μIU/mL-5.5 μIU/mL；RT3：32.5 ng/dL-66.4 ng/dL。 Comparison of the changes of perioperative thyroid hormones between VATS and TOS group. Time 1: the day before surgery; Time 2: the first day after surgery; Time 3: the second day after surgery; Time 4: the third day after surgery; Time 5: the seventh day after surgery. Normal ranges of thyroid hormones: FT3: 2.3 pg/mL-4.2pg/mL; FT4: 0.89 ng/dL-1.76 ng/dL; TSH: 0.35 μIU/mL-5.5 μIU/mL; RT3: 32.5 ng/dL-66.4 ng/dL.

## 3 讨论

正常甲状腺功能病态综合征（euthyroid sick syndrome, ESS）是指由于非甲状腺的全身性疾病、手术、禁食引起的甲状腺功能检查的异常。文献^[6, 7]^报道，约70%的住院患者尤其是外科危重患者存在ESS。ESS的甲状腺功能异常以低T3综合征最常见，占住院患者的25%-50%，其特点是血清T3水平降低，FT3水平正常或减低，FT4水平正常或升高，rT3水平升高，TSH水平多正常^[8]^。一般认为ESS是各种疾病状态下出现的一种自我保护机制，在甲状腺激素中血清T3主要参与机体的分解代谢，所以血清T3水平的降低，对于保护机体减少消耗是非常重要的。Cengiz等^[9]^则对80例病理确诊的NSCLC患者的甲状腺激素水平进行研究，共检测出28例患者（35%）发生ESS，并且病理分期越晚，ESS发生率越高，因此ESS可作为反映NSCLC病情严重程度及评估预后的预测因子。

我们研究表明，NSCLC患者行肺叶切除术后均出现FT3下降，rT3升高，FT4及TSH的变化均在正常值范围内，从而主要表现为低T3综合征。甲状腺激素中的FT3是主要的生物活性物质，肺叶切除手术导致FT3下降、rT3升高的原因考虑有：①肺叶切除手术属大手术，组织中的5’脱碘酶活性受抑制或含量下降，从而导致外周组织中T3生成减少，致使T3浓度下降。T4的内环脱碘酶被激活，T4转换为rT3增加，同时rT3分解代谢减少，血清中rT3浓度升高；②手术应激状态，机体IL-1、IL-6、TNF-α等细胞因子水平升高，这些细胞因子会抑制TRH、TSH、T3、TBG等的合成与分泌；③机体皮质醇、多巴胺等激素水平升高，进而影响下丘脑-垂体-甲状腺轴的功能。根据手术方式的不同，我们将患者分为胸腔镜组及开胸组，两组围手术期各甲状腺功能指标变化趋势均相似，但VATS组的变化幅度要小于TOS组，尤其是rT3的变化，并且上述指标恢复至术前水平的时间也要短于TOS组，进而说明，VATS手术对围手术期甲状腺激素水平的影响小，机体创伤小，应激反应轻。这与林学正^[10]^等的研究结论是相一致的。

甲状腺激素在机体三大营养物质代谢、机体免疫、心功能等方面都有非常重要的调节作用，有研究^[11]^发现ESS时血清T3、T4水平可随原发病的恶化而更趋向低下，游离T3下降常是病情严重的标志之一。NSCLC患者本身可能出现ESS，手术治疗后，手术应激会进一步加重这种异常变化，因此，围手术期监测甲状腺激素水平的变化对于肺癌患者的病情评估及预后评价有重要意义。目前对于ESS患者是否行甲状腺激素替代治疗仍有争议，但有研究^[12]^表明对心衰、CABG、心脏移植手术等的患者行甲状腺激素治疗可能会有益处，因此我们考虑，对于心功能差或者合并甲低的肺癌患者，围手术期监测甲状腺激素水平的变化对其临床治疗具有一定的指导意义。

胸腔镜手术对围手术期甲状腺激素水平影响小，这样既能让机体适应手术应激状态，减少机体消耗，又不会使甲状腺激素水平产生更大的波动，甚而影响激素功能。另外，基于存在NSCLC患者合并ESS的情况^[9]^，我们考虑VATS术后甲状腺激素水平较小的波动，可能也有利于肺癌疾病本身的治疗，改善患者预后。

综上所述，肺叶切除手术可导致机体产生正常甲状腺功能病态综合征，围手术期对甲状腺激素水平的监测可指导肺癌患者治疗、评估病情及预后；相对于传统开胸手术，胸腔镜手术后机体甲状腺素水平波动小，恢复时间短，机体应激反应小，利于肺癌患者术后恢复。
